# Sleepiness while driving and shiftwork patterns among Korean bus drivers

**DOI:** 10.1186/s40557-017-0203-y

**Published:** 2017-10-09

**Authors:** Seyoung Lee, Hyoung-Ryoul Kim, Junsu Byun, Taewon Jang

**Affiliations:** 1 Department of Occupational & Environmental Medicine, College of Medicine, The Catholic University of Korea, 222 Banpo-Daero, Seocho-Gu, Seoul, 137701 Republic of Korea; 2 Department of Occupational and Environmental Medicine, Hanyang University Guri Hospital, Seoul, Republic of Korea

**Keywords:** Sleepiness, Shiftwork, Bus drivers, Karolinska sleepiness scale, Traffic accidents, Occupational drivers

## Abstract

**Background:**

Sleepiness while driving has been regarded as a major cause of death due to traffic accidents. We compared the degree of sleepiness across five different working time periods (first, morning, post-lunch, afternoon, and last) among Korean bus drivers with different shift types (Daily two shift/Alternating day shift).

**Method:**

We interviewed 332 bus drivers with two shift types (Daily two shift, 128; Alternating day shift, 204). The questionnaire included demographic information (age, alcohol consumption and history of disease), a sleep disorder diagnosed by a doctor, job duration, the number of workdays in the past month, average working hours per workday and week, sleepiness while driving (Karolinska Sleepiness Scale), and sleeping time for both workdays and off-days. We conducted log-binomial regression analyses and produced prevalence ratios (PRs) of severe sleepiness (KSS ≥ 7) while driving with 95% confidence intervals (95% CI) to identify the difference in sleepiness for five working times between both groups.

**Results:**

For the first and morning periods, there were no statistically significant differences in the KSS scores between the two groups. However, from lunch to last driving, drivers with Alternating day shift had a much larger proportion of severe sleepiness than those on Daily two shift. Thirteen (10.2%), 2 (1.6%) and 7 (5.5%) Daily two shift workers reported severe sleepiness in the post-lunch, afternoon and last periods. In contrast, 81 (39.7%), 63 (30.9%) and 64 (31.4%) of Alternating day shift drivers experienced severe sleepiness during the post-lunch, afternoon and last driving periods (*p* < 0.0001). According to the log-binomial regression analyses, Alternating day shift was associated with severe sleepiness from lunch to last driving. After adjusting for job duration, alcohol consumption and sleeping time on workdays, the PRs were 3.97 (95% CI: 2.29–6.90) post-lunch, 18.26 (95% CI: 4.51–73.89) in the afternoon and 5.71 (95% CI: 2.51–12.99) for the last driving period.

**Conclusion:**

We found that Alternating day shift bus drivers suffered from more sleepiness while driving from lunch to last driving than Daily two shift bus drivers. This difference may be because Alternating day shift drivers had more irregular work schedules and longer working hours per day and week.

## Background

Sleepiness while driving has been regarded as a major cause of death due to traffic accidents. Out of 3583 total cases of highway accidents in 2014 in South Korea, 183 were caused by sleepiness while driving, and 38 people died, representing 13.9% of all traffic accident deaths. The percentage of deaths caused by sleepiness while driving (20.8%) was much higher than deaths due to other causes (6.9%) [1].

The prevention of sleepiness in bus drivers while driving is important because the problem is directly related to the safety of their passengers and also of pedestrians. There have been many studies about factors that could influence the sleepiness of occupational drivers. Generally, sleep disorders, including obstructive sleep apnea (OSA) [2–13], neurologic diseases [14, 15], drugs [16–18], a young age [19–22], and sleep deprivation [21–24] have been investigated as non-occupational factors that induce sleepiness while driving. Occupationally, the length of breaks [25] and long-distance driving [20, 21, 23] are known risk factors for sleepiness while driving.

A long driving time [22, 23, 26–28] is regarded as one of the causes of sleepiness while driving for occupational drivers. In 1979, the International Labor Organization (ILO) recommended that “the maximum total driving time, including overtime, shall exceed neither 9 hours per day nor 48 hours per week.” [29] Many countries, including the European Union and the United Kingdom, also limit an occupational driver’s total driving time to about 9–10 h per day to prevent cumulative fatigue and sleepiness. However, in Korea, occupational drivers (including bus drivers) are exempt from the Labor Standards Act and therefore have no limits placed on their daily driving time.

In February 2017, after the survey, the Presidential Decree and the Enforcement Regulations of the Passenger Car Transport Business Act, which guarantees a break of more than 30 min in driving for more than 4 h and a rest of at least 8 h after the end of the schedule of the day, was implemented. However, after that, a fatal accident caused by sleep driving of a bus driver who is overworked and was reported in the press.

So, we set out to determine the actual working and driving times of Korean bus drivers and also to investigate whether a long working time causes sleepiness while driving. We intended to compare their degree of sleepiness across five different work periods (first, morning, post-lunch, afternoon, and last) with different shift types (Daily two shift/Alternating day shift).

## Methods

### Study population

Seoul and Gyeonggi are the metropolitan areas with the largest population density and the heaviest traffic in South Korea. Seoul and Gyeonggi City Bus drivers have different shiftwork systems. The Seoul City Bus has a daily two-shift system (day/evening) and a rotating weekly schedule, where drivers work 8–10 h per shift for 5 or 6 consecutive days followed by 1 or 2 days off (e.g., D-D-D-D-D-Off-Off, E-E-E-E-E-E-Off; D: Day, E: Evening, O: Off). Gyeonggi City Bus has Alternating day shift system (duty/off) and a two-day rotating schedule in which drivers work for 17–20 h one day (or sometimes on three consecutive days) followed by one day off (e.g., D-Off-D-Off-D-Off-D, Off-D-D-D-Off-D-Off; D: Duty, O: Off).

We selected one Seoul City Bus company and another from the Gyeonggi City Bus system to represent each group. Among the 746 drivers who participated (Seoul: 241, Gyeonggi: 505), we interviewed 388 (Seoul: 143, Gyeonggi: 245). We excluded the 10 female drivers due to their small number. Additionally, after excluding 46 participants because of a lack of information, 332 final participants were evaluated (Seoul: 128, Gyeonggi: 204).

The study was approved by the Institutional Review Board of Catholic University of Korea at Seoul St. Mary’s Hospital (approval ID: KC15OISI0398).

### Data collection

The questionnaire survey was conducted in July 2015. We gathered information through face-to-face interviews that were carried out by trained interviewers. The questionnaire assessed demographic information, whether a person had been diagnosed with a sleep disorder by a doctor, job duration, the number of workdays in the past month, the average number of working (and driving) hours per workday and week, reported sleepiness while driving, and amount of sleeping time on both workdays and off-days. Demographic information included age, body mass index (BMI), alcohol consumption, and history of disease diagnosed by a doctor (hypertension and diabetes).

Working hours were defined as the length of time spent working from start to finish. Because most bus drivers had irregular driving times, especially due to traffic, they also typically reported irregular rest and meal times. Therefore, rest and meal times could not be excluded from their working hours. The working time of Korean bus drivers generally included rest time, refueling, car wash, etc., as well as driving time. Therefore, we investigated both working time and driving time.

We used the Karolinska Sleepiness Scale (KSS) to evaluate the degree of sleepiness while driving. We investigated the degree of sleepiness during the first driving(of day duty in the case of Daily two shift), morning, post-lunch, afternoon, and last driving(of evening duty in the case of Daily two shift), five different time periods. The KSS is a self-reported 9-point Likert scale-based questionnaire used to describe a respondent’s level of drowsiness [30]. The descriptors vary from 1 = “very alert” to 9 = “very sleepy, fighting sleep.” High (>6) KSS values are particularly associated with impaired driving performance and sleep intrusions during EEG recording [31]. A KSS score 7 or higher was defined as “severe sleepiness.”

We divided the level of alcohol consumption into three groups: non-drinker, drinking less than three times per week and drinking three times or more per week.

### Statistical analysis

We used Chi-square tests and t-tests to identify differences in demographic and other variables between Daily two shift and Alternating day shift bus drivers. We conducted log-binomial regression analyses and produced prevalence ratios (PRs) for severe sleepiness (KSS ≥ 7) while driving with 95% confidence intervals (95% CI) to identify the difference in sleepiness for the five working time periods between groups. To evaluate the effect of the variables, we used univariate and multivariate models with adjustment for covariates. Statistical analyses were performed using SAS 9.4 version (SAS institute Inc., Cary, NC, US). The level of statistical significance was set at *p* < 0.05.

## Results

Table 1 shows the demographic and work characteristics of Daily two shift and Alternating day shift bus drivers. Their mean ages were 49.8 and 48.9 years, respectively. The participants from both shifts were mostly in their 40s or 50s. Daily two shift group had a longer job duration than Alternating day shift group (13.6 years and 9.5 years, respectively (*p* < 0.0001)). The drivers from Daily two shift group had a higher proportion of overweight drivers than Alternating day shift group (Daily two shift: 43.8% and Alternating day shift: 32.4%). The level of alcohol consumption was similar in both groups. About 40% of drivers drank less than three times per week, while about 30% drank three times or more per week. The incidence rates of hypertension and diabetes were about 30% and 10% in both groups, respectively. The number of workdays in the past month in Alternating day shift group (mean: 15.1 days) was lower than that of Daily two shift group (mean = 23.6 days) because of their shiftwork characteristics (*p* < 0.0001). In contrast, the daily working hours and driving hours of Alternating day shift group (mean: 19.4 h and 17.1 h, respectively) were higher than those of Daily two shift group (mean: 10.2 h and 8.4 h) for the same reason (*p* < 0.0001). The weekly working hours and driving hours of Alternating day shift group (mean: 68.2 h and 60.0 h, respectively) were also higher than Daily two shift group (mean: 56.0 h and 46.5 h, respectively) (*p* < 0.0001).

**Table 1:** Demographic and work characteristics of Daily two shift and Alternating day shift bus drivers

Variables	Daily two shift (*n* = 128)	Alternating day shift (*n* = 204)	*p*-value
	N (%) or M ± SD	N (%) or M ± SD	
Age (years)			
< 40	14 (10.9)	29 (14.2)	<0.0001
40–49	51 (39.8)	61 (29.9)	
50–59	44 (34.4)	110 (53.9)	
≥ 60	19 (14.8)	4 (2.0)	
M ± SD	49.8 ± 8.7	48.9 ± 7.4	0.3388
Job duration (years)			
< 10	47 (36.7)	117 (57.4)	0.0012
10–20	56 (43.8)	58 (28.4)	
≥ 20	25 (19.5)	29 (14.2)	
M ± SD	13.6 ± 8.5	9.5 ± 7.2	<0.0001
Body mass index (kg/m^2^)			
< 25	72 (56.3)	138 (67.7)	0.0360
≥ 25	56 (43.8)	66 (32.4)	
Alcohol consumption			
Non-drinker	41 (32.0)	63 (30.9)	0.6450
< 3 times/week	49 (38.3)	88 (43.1)	
≥ 3 times/week	38 (29.7)	53 (26.0)	
History of disease			
Hypertension	42 (32.8)	52 (25.5)	0.1495
Diabetes	11 (8.6)	24 (11.8)	0.3598
Number of workdays in the past month	23.6 ± 1.4	15.1 ± 1.6	<0.0001
Working hours			
Daily working hours	10.2 ± 1.2	19.4 ± 1.3	<0.0001
Weekly working hours	56.0 ± 7.6	68.2 ± 7.2	<0.0001
Daily driving hours	8.4 ± 1.1	17.1 ± 1.6	<0.0001
Weekly driving hours	46.5 ± 6.3	60.0 ± 6.7	<0.0001

*M* Mean, *SD* Standard deviation

We compared sleep problems and sleepiness while driving of Daily two shift and Alternating day shift groups (Table 2). The percentages of drivers in both groups who had been diagnosed with a sleep disorder by a doctor were 6.3% and 8.8%, respectively (*p* = 0.3956). The average sleeping time of Alternating day shift drivers was lower than that of Daily two shift drivers. The average number of hours spent sleeping on workdays by Daily two shift drivers was 6.6 h, while Alternating day shift drivers slept 6.0 h (*p* < 0.0001). The average sleeping time on off-days for Daily two shift drivers was 8.2 h, while Alternating day shift drivers slept an average of 7.7 h (*p* = 0.0013). Especially on workdays, the percentage of Alternating day shift drivers who had slept less than 6 h was 36.3%.

**Table 2:** Sleep problems and sleepiness while driving reported by Daily two shift and Alternating day shift bus drivers

Variables	Daily two shift (*n* = 128)	Alternating day shift (*n* = 204)	*p*-value
	N (%) or M ± SD	N (%) or M ± SD	
Sleep disorder diagnosed by a doctor	8 (6.3)	18 (8.8)	0.3956
Sleeping time on workdays (h)			
< 6	16 (12.5)	74 (36.3)	<0.0001
6–8	86 (67.2)	106 (52.0)	
≥ 8	26 (20.3)	24 (11.8)	
M ± SD	6.6 ± 1.0	6.0 ± 1.3	<0.0001
Sleeping time on off-days (h)			
< 6	3 (2.3)	15 (7.4)	0.0014
6–8	35 (27.3)	85 (41.7)	
≥ 8	90 (70.3)	104 (51.0)	
M ± SD	8.2 ± 1.4	7.7 ± 1.6	0.0013
Karolinska sleepiness scale (KSS, M ± SD)			
First	3.3 ± 1.8	3.1 ± 1.9	0.3346
Morning	3.7 ± 1.8	3.4 ± 1.7	0.1355
Post-lunch	3.9 ± 1.8	5.4 ± 2.0	<0.0001
Afternoon	3.0 ± 1.5	5.0 ± 2.0	<0.0001
Last	3.3 ± 1.7	5.0 ± 2.3	<0.0001
Severe sleepiness while driving (KSS ≥ 7)			
First	8 (6.3)	15 (7.4)	0.7001
Morning	11 (8.6)	14 (6.9)	0.5607
Post-lunch	13 (10.2)	81 (39.7)	<0.0001
Afternoon	2 (1.6)^a^	63 (30.9)	<0.0001
Last	6 (4.7)	64 (31.4)	<0.0001

*M* Mean, *SD* Standard Deviation

^a^By Fisher’s exact test

There was no statistically significant difference in KSS scores between both groups while driving during the first time period or in the morning. But from lunch to last driving, the KSS score of Alternating day shift group was significantly higher than that of Daily two shift group (*p* < 0.0001). The mean KSS score of Daily two shift group during the first driving period of the day was 3.3. The KSS score in this group increased to 3.9 at post-lunch and then decreased to 3.3. In contrast, the KSS score of Alternating day shift group increased dramatically up to 5.4 at post-lunch and hardly recovered until the last driving period (mean KSS score: 5.0).

After dividing the participants into two groups according to severe sleepiness (defined as a KSS score of 7 or more), the results were similar. During the first driving period of the day, 8 of 128 Daily two shift bus drivers (6.3%) reported severe sleepiness. Among Alternating day shift bus drivers, 18 of 204 (8.8%) experienced severe sleepiness during the first driving period. Eleven (8.6%) of Daily two shift bus drivers and 14 (6.9%) of Alternating day shift drivers had severe sleepiness when they drove in the morning. There was no statistically significant difference in the proportion of severe sleepiness between either group for the first driving and morning periods. However, from lunch to last driving, Alternating day shift drivers had a much larger proportion of severe sleepiness than Daily two shift drivers. Thirteen (10.2%), 2 (1.6%) and 7 (5.5%) of Daily two shift bus drivers reported severe sleepiness during the post-lunch, afternoon and last driving periods. In contrast, 81 (39.7%), 63 (30.9%) and 64 (31.4%) of Alternating day shift bus drivers experienced severe sleepiness during the post-lunch, afternoon and last driving periods (*p* < 0.0001).

**Fig. 1:**
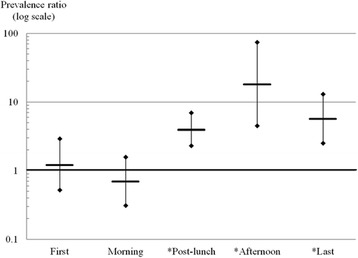
The adjusted prevalence ratios of severe sleepiness. The adjusted prevalence ratios (95% CI) of severe sleepiness (KSS ≥ 7) while driving for Alternating day shift bus drivers relative to Daily two shift bus drivers for five different time periods (Adjusted for job duration, alcohol consumption and sleeping time on workdays)

Table 3 shows crude and adjusted PRs with 95% CIs for severe sleepiness while driving for Alternating day shift bus drivers compared to Daily two shift bus drivers. Crude PRs during the first driving and morning periods were 1.18 (95% CI: 0.51–2.70) and 0.80 (95% CI: 0.37–1.70) and were not statistically significant. At post-lunch, crude PRs increased to 3.91 (95% CI: 2.27–6.72) and reached a PR peak of 19.76 (95% CI: 4.92–79.37) in the afternoon. During the last driving period, a crude PR was 6.69 (95% CI: 2.99–15.00). After adjustment for variables like job duration, alcohol consumption and sleeping time on a workday, the results were similar between all groups. After adjusting for job duration, PRs were 1.21 (95% CI: 0.52–2.82) for the first driving period, 0.77 (95% CI: 0.35–1.67) in the morning, 4.12 (95% CI: 2.39–7.11) at post-lunch, 19.66 (95% CI: 4.88–79.20) in the afternoon, and 6.36 (95% CI: 2.83–14.32) during the last driving period. After adjustment for job duration, alcohol consumption and sleeping time on a workday (Table 3 and Fig. 1), PR values were 1.22 (95% CI: 0.52–2.89) at first driving, 0.70 (95% CI: 0.31–1.57) in the morning, 3.97 (95% CI: 2.29–6.90) at post-lunch, 18.26 (95% CI: 4.51–73.89) in the afternoon, and 5.71 (95% CI: 2.51–12.99) during the last driving period.

**Table 3:** Crude and adjusted prevalence ratios (95% CI) of severe sleepiness (KSS ≥ 7) while driving for Alternating day shift bus drivers relative to Daily two shift bus drivers for five different time periods

Driving time	Crude PR (95% CI)^a^	Adjusted PR (95% CI)^b^	Adjusted PR (95% CI)^c^
First	1.18 (0.51–2.70)	1.21 (0.52–2.82)	1.22 (0.52–2.89)
Morning	0.80 (0.37–1.70)	0.77 (0.35–1.67)	0.70 (0.31–1.57)
Post-lunch	*3.91 (2.27–6.72)	*4.12 (2.39–7.11)	*3.97 (2.29–6.90)
Afternoon	*19.76 (4.92–79.37)	*19.66 (4.88–79.20)	*18.26 (4.51–73.89)
Last	*6.69 (2.99–15.00)	*6.36 (2.83–14.32)	*5.71 (2.51–12.99)

* *p* < 0.05, ^a^Unadjusted for other variables, ^b^Adjusted for job duration, ^c^Adjusted for job duration, alcohol consumption and sleeping time on workdays, *PR* Prevalence Ratio, *CI* Confidence Interval

## Discussion

We found that Alternating day shift bus drivers suffered from more sleepiness while driving from lunch to last driving than Daily two shift bus drivers, which may be because Alternating day shift workers displayed characteristics of more irregular work schedules and longer working hours per day and week. After adjusting for job duration, alcohol consumption and sleeping time on workdays, Alternating day shift was still associated with severe sleepiness while driving and showed 3.97 to 18.26 of PRs from lunch to last driving relative to Daily two shift. But it should be careful not to give undue meaning to the PR figures themselves, since KSS was a subjective questionnaire and not the medical standard.

There have been many studies about the factors associated with sleepiness while driving. In one study of commercial bus and truck drivers [28], 61% of drivers who worked longer than 12 h daily and faced long hours of driving including more than 4 h at a time reported feeling drowsiness while driving. These results are similar to the findings of our study. The very long driving hours of Alternating day shift (daily mean: 17.1 h) may be a crucial factor for the high proportion of severe sleepiness reported from lunch to last driving in this study.

In another study that administered polysomnography (PSG) to shiftwork bus drivers [32], the daytime sleep of shiftwork bus drivers was of a poorer quality than their nighttime sleep. In our study, Alternating day shift had more irregular schedules with alternately arranged workdays and off-days. Therefore, daytime sleep that occurs on off-days may be compensation for the long work time of workdays in this group. It is also possible that daytime sleeping on an off-day made drivers more tired by interrupting their circadian rhythms.

Sleep deprivation and sleep disorders [21–24] are known risk factors for sleepiness while driving. In our study, Alternating day shift had short sleeping times on both workdays and off-days. This trend may be related to their long driving and working hours and irregular working schedules. In this study, we did not adjust for whether the drivers had a sleep disorder. However, there was not much difference in this factor between the two groups, and it may be of little influence because we adjusted the participants’ sleeping time that correlated with the effect of sleep disorders. Obstructive sleep apnea (OSA) [2–13, 33] is a particularly well known major risk factor for sleepiness while driving and traffic accidents. Although we did not ask respondents about having OSA, we used their BMI as a surrogate variable because it is closely related to OSA [33, 34]. The proportion of overweight (BMI ≥ 25) drivers on Alternating day shift was lower than that of Daily two shift. Therefore, it seems that the prevalence of alternating shift drivers that had OSA was rarely higher than that of Daily two shift drivers.

A young age and short job duration [19–22, 35] were also strong risk factors for sleepiness while driving and traffic accidents. However, the mean age of Daily two shift and Alternating day shift were similar. In addition, age had a strong correlation with job duration in this study (results not shown). After adjusting for job duration, the difference in sleepiness between the two groups changed little.

Because Alternating day shift had an irregular work schedule and long driving hours [36], they were likely to be more vulnerable to cumulative fatigue than workers on Daily two shift. According to our study, cumulative fatigue does not occur in the morning but instead takes place after lunch and may cause sleepiness while driving. The lower sleeping time (both on workdays and off-days) for those on Alternating day shift may also be a factor that produces cumulative fatigue. An irregular work schedule and long working hours can cause sleep problems [37, 38] and therefore directly cause cumulative fatigue or indirectly influence cumulative fatigue to produce sleep deprivation.

Most countries regulate the daily and weekly work times of occupational drivers according to the ILO recommendations for road transport [29]. Our results suggest a lack of regulation may threaten public safety. Therefore, exceptions to the Labor Standards Act in Korea should be removed to reduce the risk of traffic accidents due to sleepiness while driving. Bus companies that still use Alternating day shift should also change this shiftwork pattern to reduce the driving time of individual employees.

To our knowledge, this study was the first to investigate the sleepiness of occupational drivers at different times of day. We discovered that severe sleepiness in the long working hours group (Alternating day shift) markedly increased after lunch. This study also verified the influence of working shift on severe sleepiness. In addition, we showed that drivers on Alternating day shift may suffer from severe sleepiness and are more likely to experience traffic accidents than workers on Daily two shift. These results were still valid after adjustment for job duration, an important confounding factor.

This study has some limitations. First, it was a cross-sectional study that used questionnaires. Therefore, the data used in this investigation may reflect recall bias because answers depended on participant memory. However, the information that this study required (e.g. degree of sleepiness while driving, driving time, working time, and number of workdays) is usually easy to recall.

Second, it is difficult to know what the definite cause is that Alternating day shift had a higher proportion of severe sleepiness. This shift had a longer daily driving (working) time due to the characteristics of shiftwork (repeating workdays and off-days by alternating days). However, the actual mean weekly driving (working) time of Alternating day shift was also higher than Daily two shift. In addition, sleeping time (both on workdays and off-days) of Alternating day shift was lower than that of Daily two shift. As a result, it is difficult to pinpoint what made drivers on Alternating day shift sleepier. It was unlikely that just one factor produced the markedly high PR for severe sleepiness in Alternating day shift drivers. Additional studies will be required to better isolate the reason behind this finding.

Third, it was difficult to evaluate the effect of a relatively lower working time on sleepiness because the working time of Alternating day shift was abnormally high. Further study is needed on the relationship between working time and daytime sleepiness in a relatively low working time condition and whether the relationship demonstrates a deterministic or stochastic trend.

Fourth, we did not sufficiently adjust for confounding factors like sleep disorders (including OSA) which could have influenced drivers’ sleepiness. Most sleep disorders are correlated with sleeping time. Therefore, we only adjusted for sleeping time instead of adjusting for sleep disorders as well. In addition, it is not problematic that other factors were not adjusted because the difference in the degree of severe sleepiness between Daily two shifts and Alternating day shift was so high.

## Conclusions

We found that bus drivers who had long driving hours and irregular shiftwork reported more sleepiness while driving. This finding may be extended to occupational drivers other than bus drivers. According to the results of this study and also the ILO recommendations for road transport, a limit for the driving time of occupational drivers should be established.
